# Purinergic Receptor Functionality Is Necessary for Infection of Human Hepatocytes by Hepatitis Delta Virus and Hepatitis B Virus

**DOI:** 10.1371/journal.pone.0015784

**Published:** 2010-12-20

**Authors:** John M. Taylor, Ziying Han

**Affiliations:** Fox Chase Cancer Center, Philadelphia, Pennsylvania, United States of America; Yonsei University, Republic of Korea

## Abstract

Hepatitis B virus (HBV) and hepatitis delta virus (HDV) are major sources of acute and chronic hepatitis. HDV requires the envelope proteins of HBV for the processes of assembly and infection of new cells. Both viruses are able to infect hepatocytes though previous studies have failed to determine the mechanism of entry into such cells. This study began with evidence that suramin, a symmetrical hexasulfated napthylurea, could block HDV entry into primary human hepatocytes (PHH) and was then extrapolated to incorporate findings of others that suramin is one of many compounds that can block activation of purinergic receptors. Thus other inhibitors, pyridoxal-phosphate-6-azophenyl-2′,4′-disulfonate (PPADS) and brilliant blue G (BBG), both structurally unrelated to suramin, were tested and found to inhibit HDV and HBV infections of PHH. BBG, unlike suramin and PPADS, is known to be more specific for just one purinergic receptor, P2X7. These studies provide the first evidence that purinergic receptor functionality is necessary for virus entry. Furthermore, since P2X7 activation is known to be a major component of inflammatory responses, it is proposed that HDV and HBV attachment to susceptible cells, might also contribute to inflammation in the liver, that is, hepatitis.

## Introduction

Hepatitis B virus (HBV) and hepatitis delta virus (HDV) are significant causes of chronic liver disease which often progresses to cirrhosis, fibrosis and hepatocellular carcinoma [1], [2]. HBV and HDV are enveloped viruses. HBV encodes three related envelope proteins and HDV, that is a subviral satellite of HBV, uses the same proteins for virus assembly and for the infection of susceptible cells. Contributing to the discovery of HDV was that it makes HBV infections more damaging [3].

HBV and HDV infections target hepatocytes in the liver. Experimentally, primary cultures of hepatocytes can be infected by both viruses and it is considered that both may use the same or similar mechanisms to achieve entry [4]. Studies over many years have reported a variety of candidate host receptors for the infection but none have been confirmed or established [4].

In 1988 we reported that suramin, a symmetrical hexasulfated napthylurea, was able to block the infection of primary woodchuck hepatocytes by HDV [5]. Moreover, it blocked infection of primary duck hepatocytes by duck hepatitis B virus, a relative of HBV. More recently, others have shown that suramin can block infection by HBV [6]. Suramin has been demonstrated to block infections by other animal viruses [7], [8], [9]. It blocks infection of liver cells by *Plasmodium falciparum* sporozoites, and has been used clinically to treat trypanosomiasis and filariasis [10], [11].

Seemingly independent of these effects of suramin on infections, others have discovered that it is an antagonist of purinergic receptors [12]. Numerous such receptors have been characterized and studied largely for their roles in neuronal signaling although other studies have detected their presence on many cell types, such as monocytes and muscle cells [13]. There are seven P2X receptors, all of which are ligand-gated cationic receptors, which in nature respond to extracellular ATP. They are sequence-related and structurally have two trans-membrane domains and an extracellular loop containing essential cysteine cross-links and five N-linked glycosylation sites [14]. P2X7 differs from the others in that it contains a significant (220 amino acid) C-terminal cytosolic extension that interacts with at least 11 identified host proteins [15] and is responsible, upon activation, for the transmission of many membrane trafficking responses [16]. Chronic activation of P2X7 can produce apoptosis and thus not surprisingly, expression and activation of this receptor is tightly regulated.

Activation of some purinergic receptors by ATP or non-natural agents such as BzATP can be blocked by suramin. Other blockers include pyridoxal-phosphate-6-azophenyl-2′, 4′-disulfonate (PPADS) [17] and brilliant blue G (BBG) [18]. BBG is more specific for just P2X7 [19], [20], [21], and because of the awareness of the importance of P2X7 in processes such as cytokine release, inflammatory and neuropathic pain and renal fibrosis [21], there has been a major effort to develop more specific and potent inhibitors [19], such as AZ11645373 [22]. As documented here we tested compounds in addition to suramin for their effect on HDV and HBV infection of primary human hepatocyte (PHH) cultures. PPADS and BBG were inhibitory, leading us to assert that the functionality of one or more purinergic receptors is essential for virus entry. And given the reported specificity of BBG [19], [20], we would suggest that activation of P2X7 in particular, is a necessary component of virus entry into susceptible cells. This novel finding has many implications for understanding host cell entry by these and perhaps other infectious agents.

## Results

These studies were begun with HDV rather than HBV for two reasons. First, HDV reaches maximal replication in PHH by 6 days, compared to 12 for HBV; based on our observations of the limited viability of the primary human hepatocytes cultures, HDV was therefore preferable. Second, for HDV we assay for antigenomic RNA (the exact complement of the genomic RNA, which is not present in virus, and only appears in cells as a result of the infection process) and this has a far greater sensitivity to noise ratio than assays for HBV.

As mentioned in the Introduction it was known that HDV infection of primary woodchuck hepatocytes could be blocked by suramin at 70 µM [5]. Therefore one initial aim was to determine whether this inhibition applied to the more relevant infection of PHH, and to compare suramin action with a synthetic preS1 peptide at 50 nM. This peptide was shown by Urban and colleagues to act as a potent inhibitor of HDV infection [4], a finding we have confirmed [23]. Thus, a second aim was to compare the effects of suramin and preS1 peptide when added at different times relative to a 3-hour exposure of PHH to HDV. A third aim was to test in parallel heparin, which has been reported to block infection by HBV [6], [24].

As summarized in Table 1, all three compounds were found to inhibit HDV infection when present from −1 to +16 h relative to the 3-hour virus exposure. When present only before and during the virus exposure the inhibition was significantly less. And yet, when present immediately after the virus exposure, there was still significant inhibition. Addition of the compounds at 16 h after virus exposure had very little effect, which argues against toxicity. Thus that observation that the inhibitors had a potent effect when added immediately after virus exposure indicates that entry was slow with less than 50% of the virus that attached within 3 h, as having been able to achieve entry in that time. Further it raises the question as to how preS1 peptide and suramin, and to a lesser extent heparin, were able to act at a step needed after attachment, that is, at virus entry.

**Table 1:** Inhibition of HDV infection by preS1 peptide, heparin, and suramin.

Time when inhibitor present (hours)	% HDV replication in presence of inhibitor		
	PreS1 peptide	Heparin	Suramin
−1 to +16	3.1±1.2	9±5	1.4±0.7
−1 to +3	65±19	24±10	9±7
+3 to +16	12±4	43±11	21±8
+16 to +40	82±33	72±14	75±16

PHH in HGM were exposed to HDV for 3 h. PreS1 peptide (50 nM), heparin (100 µg/ml) or suramin (70 µM) were present for the indicated periods of time. After removal of unattached virus with two washings at +3 h, infections were allowed to proceed out to 6 days at which time total RNA was extracted and analyzed by qPCR for antigenomic RNA. Data are expressed relative to cells not treated with inhibitors, along with the standard error of the mean.

As described in the Introduction another activity of suramin is as an inhibitor of purinergic receptors [12], [13]. Thus we tested the inhibitory ability of suramin relative to two other antagonists: PPADS and BBG. In these tests the HDV was again present for 3 h and the inhibitors, at a series of concentrations, were present from −1 to +3 h. In all cases, to eliminate consideration of virus that attached but only entered after the 3-hour exposure period, preS1 peptide was added from +3 to +16 h, and infection was assayed at 6 days.

**Figure 1:**
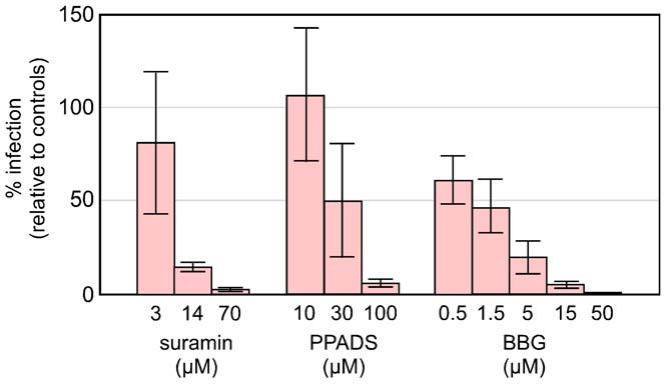
Inhibition of HDV infection by suramin, PPADS and BBG. PHH were exposed to HDV in HGM for 3 h. From −1 to +3 h the inhibitors were also present, at the concentrations indicated. At +3 h both inhibitors and virus were removed and replaced by media containing preS1 peptide (50 nM) for the next 16 h, after which the cells were incubated in HGM out to 6 days, at which time total RNA was extracted and assayed by qPCR for HDV antigenomic RNA. As described in Methods, the mean values obtained are expressed relative to untreated control cultures. Error bars represent calculated standard error of the mean.

As summarized in Fig. 1 suramin, PPADS and BBG, each gave significant inhibition of HDV infection. To exclude the post-entry effect of cell toxicity two controls were used. First, just as shown in Table 1, when the inhibitors were added 16 h after virus exposure, no significant inhibition was detected. Thus even a 24-hour exposure administered after virus entry, did not interfere with virus replication. Second, infections were performed with another virus, namely with a vesicular stomatitis virus, VSV that expressed GFP. This virus is reported to be rapidly internalized by clathrin-mediated dynamin-2-independent endocytosis [25]. Cells were exposed to both VSV and compound for 16 h, at which time infection was monitored by fluorescence microscopy. At the highest concentrations of these three compounds there was no detectable effect on the extent of VSV infection and replication (data not shown). Together, these two control studies support the interpretation that suramin, PPADS and BBG block HDV infection via an inhibition of an essential purinergic receptor function.

As mentioned in the Introduction there are reports that BBG, unlike suramin and PPADS, is more specific for a single purinergic receptor, P2X7. This particular receptor is unique relative to the others and has many interesting roles, especially relating to inflammation and signaling of pain. Recently this has led many to search for inhibitors that are even more specific and more potent [19]. AZ11645373 has been characterized as a potent non-competitive, slowly reversible, and highly specific antagonist of human P2X7 [22], [26]. At 10 µM this compound reduced HDV infection of PHH to 15±7% (data not shown). However, this treatment led to demonstrable cell toxicity in PHH. Also it totally blocked the ability of VSV to infect PHH. We favor the interpretation that this inhibitor was too inhibitory and/or specific when used under our experimental conditions. In this respect we note that many of the newer P2X7 inhibitors have been tested in short term experiments, with exposure to cells often for periods of only minutes, and in specifically defined media conditions. In contrast, BBG has been tested on cultured cells and even in vivo, with effects that are considered specific for P2X7 [27].

In the study summarized in Fig. 1 preS1 peptide was added at +3 hr to block entry of virus that had attached but failed to enter within the 3-hour virus exposure period. In the following experiments, an alternative strategy was used that did not require use of preS1 peptide. A modification was to expose PHH to virus, with and without inhibitor, for the longer period of 16 h, so as to allow a greater level of both virus attachment and entry. A second change was to include a test of HBV relative to HDV. Prior studies indicated that for some unclear reasons, HBV infections of PHH were less efficient than HDV, and so the longer infection time was advantageous.

**Figure 2:**
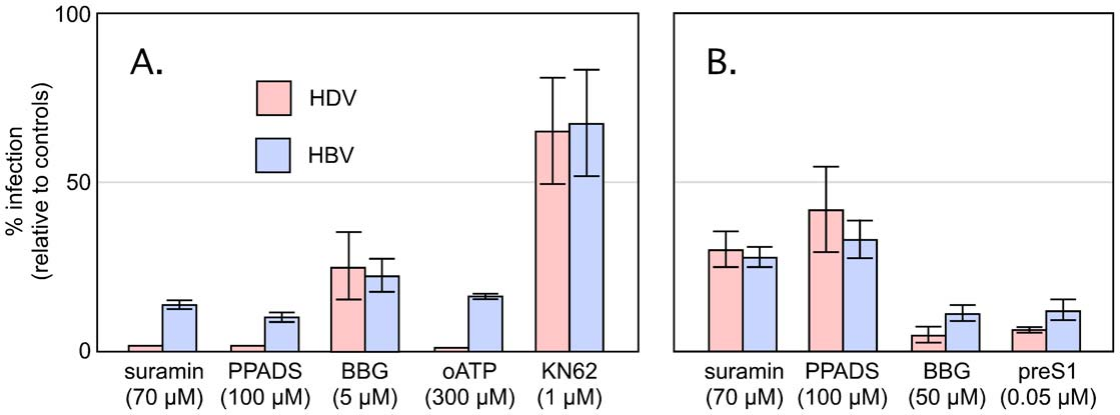
Inhibition of HDV and HBV infections by inhibitors of purinergic receptors. PHH in HGM were exposed to HDV (red) or HBV (blue) for 16 h in the absence (panel A) or presence (panel B) of 5% PEG, along with the indicated inhibitors and their concentrations. After 16 h media was changed to HGM and the infections allowed to continue for 6 or 12 days, for HDV and HBV, respectively, at which times total RNA was extracted and assayed by qPCR as described in Methods. Evaluation was as in Fig. 1.

As shown in Fig. 2A, it was found that suramin, PPADS and BBG inhibited infection by both HDV and HBV. The extent of infection for HDV was similar to that obtained in Fig. 1, except that now the preS1 peptide was not used. The inhibition for HBV was extensive but maybe not as much as for HDV.

In addition we tested the effect of oxidized ATP, oATP, a potent antagonist of P2X7 that acts by irreversible binding to the ATP-binding site [28]. As shown, oATP at 300 µM blocked both HDV and HBV infections. This concentration is reported to be specific for P2X7 [20]. However, in parallel controls it completely blocked VSV infection of PHH. Also tested was KN62, a molecule first developed as a specific cell-permeable inhibitor of the autophosphorylation of Ca^++^/calmodulin-dependent protein kinase II. It is considered to inhibit calcium mobilization after activation of human P2X7 receptors [19]. However, it had no significant effect on HDV or HBV.

Others and we have reported that 4 to 5% polyethylene glycol, PEG, present during the attachment and entry, can greatly enhance the extent of HDV infection by at least 15-fold [29], [30] and others using HepaRG cells, routinely use 4% PEG for enhancing infections with HBV [31]. We repeated the experiments of Fig. 2A in the presence of 5% PEG during the 16 h infection, with results as shown in Fig. 2B. Note that the suramin and PPADS inhibited but not as much. The BBG inhibition now needed the higher concentration of 50 µM. As a reference we tested the preS1 peptide. Again there was inhibition but not as much as in the absence of PEG (Table 1). Thus it would seem that PEG allows an increase in the extent of infection but with some loss in the efficiency of the inhibitors.

## Discussion

Overall, these studies support the assertion that purinergic receptor functionality is essential for the process of PHH infection by HDV and HBV. Suramin, PPADS and BBG are known antagonists of one or more purinergic receptors [13], [32], [33] although BBG is more specific for P2X7 [19], [20]. While our data are not sufficient to prove P2X7 is involved it is worth noting that P2X7 is not only structurally different from the other six P2X receptors but its activation has many consequences, some of which could contribute to virus entry. Activation is associated with but not limited to [13], [16], [32], [34]: (i) prolonged opening of an ion channel with influx of calcium and sodium ions, (ii) formation of pores in the cell membrane, (iii) activation of caspases, (iv) activation of receptor tyrosine kinases, (v) temporary reorganization of the actin cytoskeleton, (vi) transient membrane blebbing, and (vii) fusion between cells with activated P2X7. It is known that (v) and (vi) are requirements for macropinocytosis [35]. At this time a total of 12 different viruses have been recognized as using a form of macropinocytosis as a means of entry into susceptible cells [35].

Purinergic receptors are known to assemble homo- and hetero-multimeric complexes in the plasma membrane [21]. Even if purinergic receptor functionality is necessary for the infection, further experiments would be needed to determine whether it is a direct or indirect requirement. For example, a direct effect would be if the virus binding to such a receptor complex actually causes the activation whereas an indirect effect would be if a controlled level of activation must be maintained for the virus entry even though entry occurs at a quite different site.

As mentioned in the Introduction, we have yet to identify the putative receptor for HDV and HBV. The inhibitor studies reported here only support the interpretation that both viruses use a similar mechanism [4]. The mechanism for HDV is shown here to be slow. As shown in Table 1 both the preS1 peptide and suramin can have a potent effect on virus entry even when added after the virus has attached.

Further studies are needed to establish and clarify the role of P2X7 in HDV and HBV entry. Exogenous expression of human P2X7 cDNA sequences has been previously achieved in the 293T cell line [33]. We repeated such expression in 293T, but it did not confer susceptibility to HDV infection (data not shown). However, there are associated complications. These include P2X7 expression and function vary between tissues and with species [33], human P2X7 exists in at least 7 different spliced forms [36], and there are many genetic polymorphisms [37]. Even more, the majority of the protein is often present in the cytosol rather than at the cell surface [38]. And for stability and functionality at the cell surface there are requirements for hetero- and homo-multimerization [21], as well as a requirement for extensive N-linked glycosylation [14]. Thus, appropriate expression and functionality of P2X7 could be a major contributor to the specific targeting of HDV and HBV to human hepatocytes.

The observation that suramin, PPADS and BBG give inhibitions very similar to the preS1 peptide and even to heparin needs to be explained. We previously reported that when preS1 sequences either alone or fused to the N-terminus of an immunoglobulin heavy chain, are expressed by transfection of cells, the proteins are efficiently secreted [23]. This shows that the preS1 region can act as an uncleaved signal peptide, and is consistent with observations that synthetic preS1 peptide can make efficient interactions with hepatocyte membranes [39]. Others have shown that suramin can block entry of a malarial parasite by inhibiting secondary processing of the N-terminal signal sequence of an essential surface protein [11]. Therefore, a speculation is that post-attachment processing of preS1 domains on the surface of HDV and HBV is essential for virus entry, and that this processing can be inhibited by heparin and preS1 peptide as well as inhibition of purinergic receptor functionality.

If the attachment of HDV and HBV to hepatocytes does in fact activate P2X7, even transiently, this could be linked to the knowledge that P2X7 is a regulator of several key inflammatory molecules, including IL-1β, IL-18, TNF-alpha and IL-6 [40]. Thus, the attachment of virus to hepatocytes, and maybe also to non-hepatocytes [41], such as Kupffer cells in the liver, could contribute to an inflammatory response, in this case, hepatitis. We have detected in PHH by immunoblot a 60 kDa band consistent with P2X7 expression (data not shown). Others have reported hepatocyte P2X7 protein as detected by immunostaining and mRNA detected by northern [41], [42]. Inflammation in the liver can also be mediated by P2X7 activation [43]. Thus our findings allow the speculation that HBV and HDV might contribute to liver damage, damage that might be addressed using specific P2X7 antagonists [44]. While it is not uncommon that patients with a high load of HBV show no signs of liver inflammation [1], it is also true that HDV titers reach levels at least 100-fold that of HBV and that HDV coinfections are much more damaging that HBV alone [2].

## Materials and Methods

### Materials

Suramin was obtained at originally described [5]. Heparin, PPADS, BBG, KN62, oATP and AZ11645373 were obtained from Sigma. The chemically synthesized preS1 2–48 myristoylated peptide (genotype D, subtype *ayw*) was a gift from Stephan Urban [45].

### Viruses

Assembly of HDV was as previously described [30]. Briefly, Huh7 cells [46] were transfected with a plasmid to initiate HDV genome replication and a plasmid to express the envelope proteins of HBV (genotype A, subtype *adw2*). Media harvested from days 6–8, 8–10, and 10–13 was clarified by low speed centrifugation. The virus was precipitated by 10% PEG, collected by centrifugation and resuspended in serum-free media, using one hundredth of the original volume. Aliquots were stored at −70°C. The source of HBV was the HepAD38 system developed by Ladner et al. [47]. These cells contain an integrated HBV sequence with expression under TET-off control. As for HDV, the tissue culture fluids were collected, clarified and then concentrated by PEG precipitation. Vesicular stomatitis virus (VSV) with an added gene for GFP, was provided by Siddarth Balachandran.

### Primary human hepatocytes

Primary human hepatocytes (PHH) in a 48-well format, plated on rat tail collagen, were obtained from Cellzdirect or Celsis, and maintained on the recommended hepatocyte growth medium (HGM), with supplements but not including fetal calf serum.

### Infections

Infection of one well of a 48-well plate used 5 µL of concentrated virus in 0.2 ml HGM. In practice the observed infection efficiencies were around 0.1–0.5%, using immunostaining for delta antigen to detect HDV infected cells at 6 d after infection. As indicated, in some experiments 5% PEG was added during 16 h exposure of cells to virus (and inhibitor), a strategy that increased the number of infected cells by at least 15-fold [30]. In another strategy (Fig. 1), cell were exposed to virus (and inhibitor) for only 3 h, after which media was removed and cells washed and then incubated for 16 h with media containing 50 nM of a synthetic peptide containing the N-terminus of the preS1 region of the large HBV envelope protein. This peptide acts as an inhibitor of further entry of virus into susceptible cells ([45], [48] and unpublished observations). All infections were performed in at least triplicate except for control infections where six separate culture wells were infected. Media was changed every 2–3 days. Typically infections extended for 6 and 12 days for HDV and HBV, respectively.

### Assays of virus infection

At the end of the infection period media was removed and immediately replaced with 0.5 ml of Tri Reagent (Molecular Research Center). RNA was then extracted according to manufacturer's instructions except that 10 µg of carrier dextran was added to facilitate the ethanol precipitation steps. Aliquots of RNA were DNase-treated immediately prior to qPCR assays to avoid what might be plasmid DNA surviving from the HDV assembly. HDV assays were directed against the antigenomic RNA of HDV, a species not detectably present in HDV particles, using primers as described in Gudima et al. [30]. HBV assays were designed to detect pregenomic RNA as described by Loeb et al. [49]. The qPCR data were averaged and the standard deviation of the mean determined. The mean percentages shown are expressed relative to the untreated control samples as 100%, and the error bars represent compounding errors for treated and untreated samples. VSV infections were monitored after 16 h of exposure of cells to an MOI 0.01 in the absence or presence of compound.
